# The SARS-CoV-2 induced targeted amino acid profiling in patients at hospitalized and convalescent stage

**DOI:** 10.1042/BSR20204201

**Published:** 2021-03-10

**Authors:** Junfang Wu, Mingming Zhao, Chenze Li, Yuxuan Zhang, Dao Wen Wang

**Affiliations:** 1 Division of Cardiology, Department of Internal Medicine, Tongji Hospital, Tongji Medical College, Huazhong University of Science and Technology, Hubei Key Laboratory of Genetics and Molecular Mechanisms of Cardiological Disorders, Wuhan 430030, China; 2 The Institute of Cardiovascular Sciences and Institute of Systems Biomedicine, School of Basic Medical Sciences, Key Laboratory of Molecular Cardiovascular Sciences of Ministry of Education, Health Science Center, Peking University, Beijing 100191, China

**Keywords:** amino acids, convalescent stage, COVID-19, SARS-CoV-2

## Abstract

The severe acute respiratory syndrome coronavirus 2 (SARS-CoV-2) has induced an ongoing global health crisis. Here we utilized a combination of targeted amino acids (AAs) and clinical biochemical profiling to analyze the plasma of coronavirus disease 2019 (COVID-19) subjects at the hospitalization stage and 1-month post-infection convalescent stage, respectively, to investigate the systematic injury during COVID-19 disease progress. We found the virus-induced inflammatory status and reduced liver synthesis capacity in hospitalized patients, which manifested with increased branched-chain AAs (BCAAs), aromatic AAs (AAAs), one-carbon related metabolites, and decreased methionine. Most of these disturbances during infection recover except for the increased levels of medium-chain acylcarnitines (ACs) in the convalescent subjects, implying the existence of incomplete fatty acids oxidation during recovery periods. Our results suggested that the imbalance of the AA profiling in COVID-19 patients. The majority of disturbed AAs recovered in 1 month. The incomplete fatty acid oxidation products suggested it might take longer time for convalescent patients to get complete recovery.

## Introduction

Severe acute respiratory syndrome coronavirus 2 (SARS-CoV-2) is the causative agent of the ongoing pandemic coronavirus disease 2019 (COVID-19). In addition to the directly damaged pulmonary type II alveolar cells resulting from the SARS-CoV-2 virus [1], unrestrained inflammatory cell infiltration can mediate damage in the lung or liver through excessive secretion of proteases and reactive oxygen species [2]. Till now, the incidence of liver injury has reached 60% in COVID-19, mainly indicated by abnormal levels of ALT/AST accompanied by slightly increased bilirubin [3–5]. Huang et al. found serum hypoalbuminemia could predict poor prognosis of COVID-19 [6]. However, there still lacks comprehensive metabolic profiling that contributed to liver dysfunction in COVID-19 subjects, and the metabolic dysfunction in convalescent subject post-SARS-CoV-2 infection remains unknown.

Amino acids (AAs), which are mainly synthesized in the liver, play an important role in the immune system and redox status except for its benefits of energy supplement [7,8]. Circulating levels of most AAs could be influenced by the catabolic conditions as severe infection or injury. Branched-chain AAs (BCAAs) have been investigated for decades as agents for enhancing muscle protein synthesis during exercise training or in aging [9,10]. Furthermore, the catabolic products of BCAAs, acylcarnitines (ACs), result from incomplete fatty acid oxidation, could provide a shuttle mechanism for acyl-coenzyme A between different subcellular organelles and give insight in the BCAA catabolism ability [11]. Secondly, the roles of glutamine, arginine, methionine and cysteine (Cys) in enhancing the immune function have been well established [12]. Because the availability of cysteine is a major factor that limits the synthesis of glutathione [13], its precursor is also highly effective in enhancing immunity under various disease states [14]. Thirdly, other AAs (such as serine and glycine) and metabolites involved in the one-carbon cycle (such as choline and trimethyllysine (TML)) could provide substrates for methylation reaction, maintaining redox status, and satisfying many requirements for essential lipids/nucleotides/proteins biosynthesis [15,16].

The objective of this work is to assess the metabolic AA profile and clinical biochemical disturbance associated with COVID-19 by targeted liquid chromatography tandem mass spectrometry (LC-MS). The alterations in the hospitalized subjects were also verified in convalescent subjects 1-month post-SARS-CoV-2 infection. Such a combination of the metabolic and clinical index in the different stages of disease progression will provide a more comprehensive understanding of virus-induced dysfunction in COVID-19 patients.

## Materials and methods

### Study cohorts and samples collection

This was a retrospective study. We obtained the medical records for hospitalized Covid-19 patients (COVID-H) between 5 February 2020 to 20 April 2020 at Tongji Hospital of Tongji Medical College, Huazhong University of Science and Technology, Wuhan, China. The demographical characteristics of all subjects were summarized in Table 1. A total of 45 samples (*n*_plasma_=15, *n*_serum_=30) collected from 28 patients in hospitalized group were divided into two aliquots: one part for clinical biochemistry tests and the other part was kept for metabolic measurement.

**Table 1:** Demographical characteristics of the COVID-19 patients at the hospitalized and recovery stages

Variables	Control (*n*=48)	COVID-H (*n*=28)	COVID-R (*n*=21)
**Sex-*n* (%)**			
Male	17 (35.4)	4 (21.4)	7 (33.3)
Female	31 (64.6)	24 (78.6)	14 (66.7)
**Age-years**			
Mean ± SEM	55.69 ± 1.69	64.67 ± 3.06	38.38 ± 1.91
Median (IQR)	63 (40–64)	67 (51–79)	38 (31–44.5)
Range	37–67	27–92	25–60
**Time from admission to discharge, days**			
Mean ± SEM		43.5 ± 4.67	16.9 ± 1.67
Median (IQR)		36 (22–66)	15 (12–23.5)
Range		15–89	2–29
**Disease severity-*n* (%)**			
Non-severe		6 (21.4)	19 (90.5)
Severe		22 (78.6)	2 (9.5)
**Disease history-*n* (%)**			
Diabetes	1 (2.1)	6 (21.4)	0 (0)
Hypertension	9 (18.8)	12 (42.9)	0 (0)
Coronary heart diseases	4 (8.3)	4 (14.3)	0 (0)
Stroke	0 (0)	4 (14.3)	0 (0)
Cardiomyopathy	0 (0)	3 (10.7)	0 (0)
Hepatitis	2 (4.2)	0 (0)	0 (0)
COPD	0 (0)	4 (14.3)	0 (0)

Abbreviations: COPD, chronic obstructive pulmonary disease; IQR, interquartile range; SEM, standard error of mean.

The recovered samples were collected from 21 COVID-19 subjects that willing to have a 1-month return visit to hospital (average days: 28.27 ± 2.08). The 48 control subjects from a previous epidemiological survey cohort without SARS-CoV-2 infection were matched with the hospitalized and convalescent COVID-19 patients from age and sex. The subjects with fever (body temperature higher than 37.3 degrees) and higher white blood cell (>9.5 × 10^9^/l) were excluded for controls.

The study was approved by the Research Ethics Committee of Tongji Medical College, Huazhong University of Science and Technology, Wuhan, China with written informed consent from all participants.

### Clinical biochemistry tests

The plasma biochemistry parameters were measured in the biochemistry laboratory at Tongji Hospital of Tongji Medical School using the Sysmex XE-2100 automatic hematology analyzer. These parameters included routine blood index, liver function tests and kidney function tests for all subjects. Besides, a panel of the cytokines, including interleukin (IL)-1β, IL-2γ, IL-6, IL-8, IL-10, and tumor necrosis factor-α (TNF-α), were measured using enhanced chemical luminescence method on IMMULITE-1000 (SIEMENS, Germany), an automated immunoassay analyzer for assessing the inflammatory status towards COVID-19 in hospitalized and convalescent patients.

### Targeted metabolic analysis for metabolite quantification

All plasma/serum samples (*n*=84 for plasma and *n*=30 for serum) were inactivated and sterilized at 56°C for 30 min before metabolic profiling [17]. The targeted metabolomics profiling was performed to measure the concentration of 37 metabolites in plasma or serum samples of the COVID-19 subjects by targeted liquid chromatography tandem mass spectrometry (SCIEX AB, QTRAP4500, UPLC-MS/MS system) [18,19]. The metabolites were identified by comparing with the retention time and the multiple reaction monitoring mass data from their corresponding internal standards. And each metabolite was quantified by comparing with the signal integrals from internal standards. Of them, 21 metabolites were calculated based on their corresponding internal standard to get the absolute concentration and 16 metabolites were calculated based on their similar internal standard to get the relative concentration. All the standards for targeted metabolites and their internal standards are listed in Supplementary Table S1. The LC gradient condition and targeted MS instrument parameters are listed in Supplementary Table S2. To take consideration of the difference between plasma and serum, we compared the targeted metabolites in plasma and serum from the same subject (*n*=15) to investigate the effects of sample type on the detected metabolites.

### Statistical analysis

The concentration values of each clinical biochemistry and metabolite index were expressed as mean ± SEM. The statistical analysis was performed by using SPSS software (Version 20, U.S.A.) and GraphPad Prism software (Version 7, U.S.A.). The one-way ANOVA with the original false discovery rate (FDR) method of Benjamini and Hochberg correction was used for multiple comparisons. Otherwise, the Kruskal–Wallis test with FDR correction was used for the non-parametric dataset. The results were considered significant when the adjusted *P*-value was less than 0.05.

Separately, the multinomial logistic regression models with FDR correction were conducted to examine cross-sectional associations of each variable with baseline status (such as gender, age, disease history) among COVID-H, COVID-19 subjects at the recovery stage (COVID-R), and their corresponding controls.

Besides, the multivariate data analysis, including the principal component analysis (PCA), Partial Least-Squares-Discriminant Analysis (PLS-DA) and Orthogonal Partial Least-Squares-Discriminant Analysis (O-PLS-DA), were used for extracting information from the dataset with multiple metabolic and biochemical variables simultaneously. All constructed models were validated by the permutation test and cross-validation ANOVA (CV-ANOVA). The figures were plotted under the R environment (Version 3.5.2).

## Results

### Demographic and general characteristics

A total of 45 samples from 28 subjects in a group of hospitalized COVID-19 (COVID-H), 21 subjects at 1-month recovery stage following SARS-CoV-2 infection (COVID-R), and 48 control samples were included in the present study (Table 1). The average age in the COVID-H group was 64.67 ± 3.06 years, and 24 (78.6%) were female. Of these patients, 21 out of 28 (75%) subjects had at least one certain fundamental disease. The average hospitalized days from admission to discharge were 43.5 ± 4.67 for COVID-H patients. The 22 (78.6%) subjects were diagnosed as severe according to the diagnostic criteria. Compared with the COVID-H group, the average age in the COVID-R group was 38.38 ± 1.91 years (*P*<0.001) (14 [66.7%] for female) and 19 [90.5%] diagnosed as mild during their hospitalization. The hospitalized days (16.9 ± 1.67) were shorter than the days in the COVID-H group. For the 48 subjects in the control group, 32 subjects were chosen for well-matched with the hospitalized patients from age and sex (Control-Old, average age: 64.25 ± 0.30, 24 (75%) were female), and 16 subjects were matched with the convalescent patients (Control-Young, average age: 38.5 ± 0.18, 7 (43%) female).

### Clinical biochemistry profiling of COVID-19 patients in hospitalization and convalescence period

**Figure 1:**
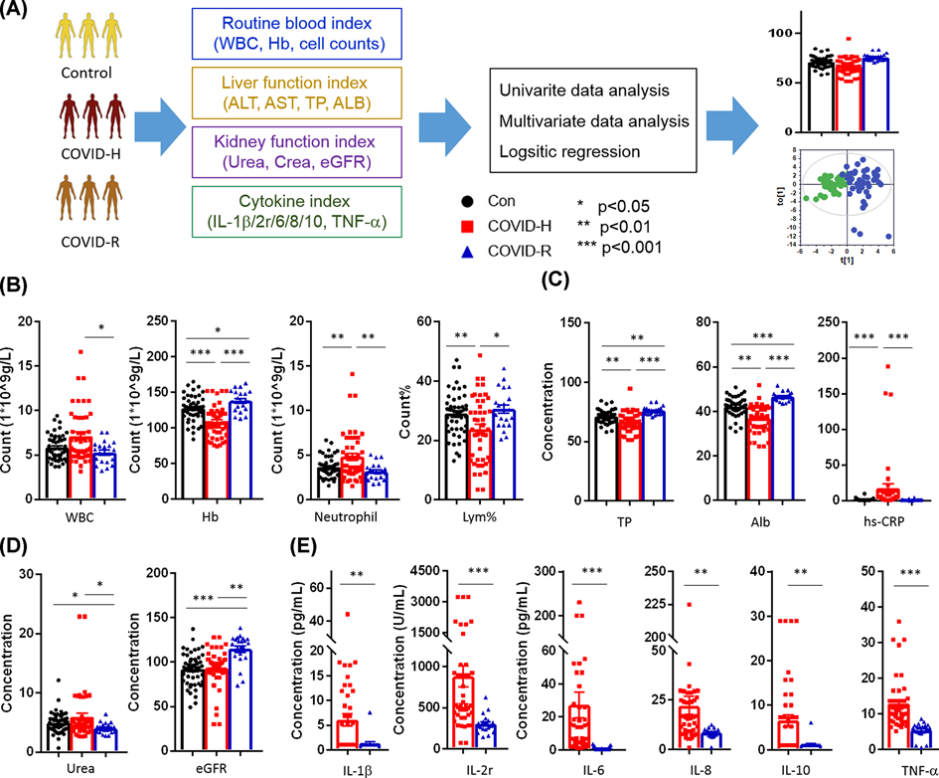
Clinical biochemistry alteration of COVID-19 patients in hospitalized and convalescent period (**A**) A brief diagram for analyzing kinds of clinical tests. (**B**–**E**) Changed biochemical tests for routine blood index, liver function index, kidney function index, and cytokines of three groups. Keys: Con, Control (●); COVID-H, hospitalized subjects of COVID-19 (▓); COVID-R, Recovered subjects of COVID-19 (▲); WBC, white blood cell; Hb, Hemoglobin; Lym%, percentage of lymphocyte; hs-CRP, hyper sensitive-C Reaction Protein; eGFR, estimated glomerular filtration rate; TNF-α, tumor necrosis factor-α. All values were represented as mean ± SEM. Statistical significance was evaluated by and one-way ANOVA test (A–C) and unpaired *t* test (D) with FDR correction. *P<0.05, **P<0.01, ***P<0.001.

According to the analysis flowchart in Figure 1A, the COVID-19 patients in the hospitalization showed stimulated inflammatory responses, including higher levels of C-reaction protein (CRP), neutrophil counts, and a lower percentage of lymphocyte (Figure 1B,C, Supplementary Table S3). The decreased hemoglobin levels suggested the anemia caused by the SARS-CoV-2 infection. Besides, the decreased levels of total protein (TP) and albumin (ALB) in the hospitalized patients implied the impaired liver ability to synthesize proteins (Figure 1C). The levels of ALT and AST had an elevated trend in hospitalized patients, even they did not reach the statistical significance for differentiating three groups (Supplementary S3). The slight changes in urea and eGFR implied that the virus affects the renal function mildly. More importantly, most of the clinical biochemistry index, which manifested by routine blood index, hepatic function index, and cytokines (Figure 1B–E), of the COVID-19 patients recovered to normal in the patients after a 1-month convalescent period.

To eliminate the effects of confound factors (including disease history, age, and sex), the statistical significance was adjusted by FDR-correction using multinomial logistic regression in Supplementary Table S4. When compared the biochemical profile between COVID-H and controls, we discovered a significant decrease in the levels of TP (*P*=0.002, OR = 0.75) and ALB (*P*=0.003, OR = 0.75), together with an increase in the levels of inflammatory factors (CRP, *P*=0.003, OR = 1.83). In the comparison between COVID-H and COVID-R, we found a significant decrease in the levels of TP/ALB and cytokines (IL-1β/6/8/10 and TNF-α) in the COVID-R.

### Targeted metabolic profiling alterations of COVID-19 patients in hospitalization and convalescence period

**Figure 2:**
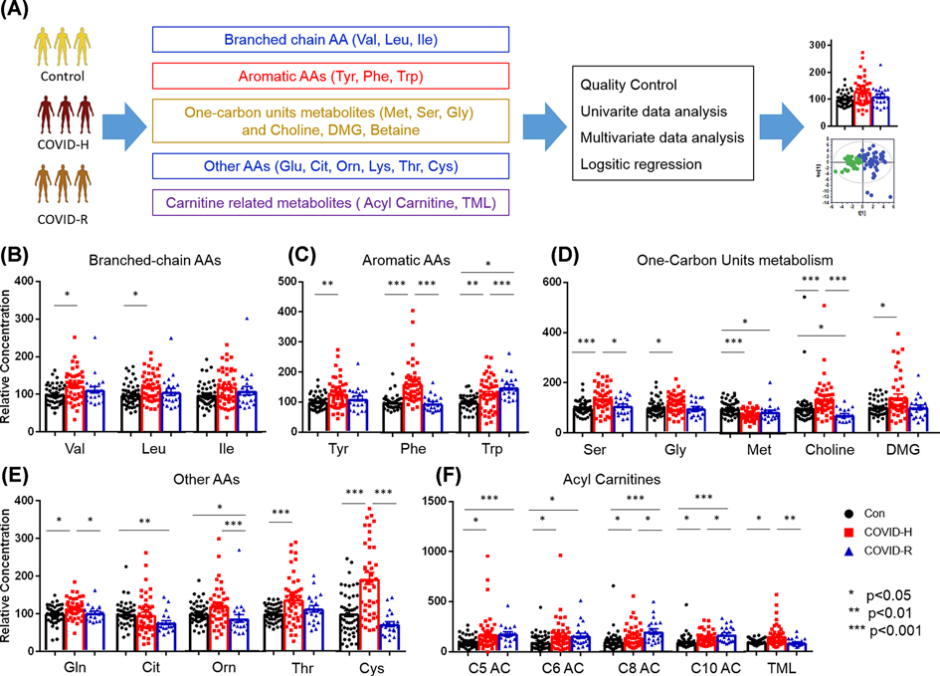
Metabolic profiling alterations of COVID-19 patients in hospitalized and convalescent periods (**A**) A brief diagram for analyzing kinds of metabolites. (**B**–**F**) Changed metabolic analysis for BCAAs, AAAs, one-carbon unit metabolites, other AAs, and carnitine-related metabolites of three groups. Keys: Con, Control (●); COVID-H, hospitalized subjects of COVID-19 (▓); COVID-R, Recovered subjects of COVID-19 (▲); Val, valine; Leu, leucine; Ile, isoleucine; Tyr, tyrosine; Phe, phenylalanine; Trp, tryptophan; Ser, serine; Gly, glycine; Met, methionine; DMG, Dimethylglycine; Gln, glutamine; Cit, citrulline; Orn, Orthinine; Thr, threonine; Cys, cysteine; AC, acylcarnitine; TML, trimethyllysine. All values were represented as mean ± SEM. Statistical significance was evaluated by one-way ANOVA test with FDR correction. *P<0.05, **P<0.01, ***P<0.001.

Targeted metabolic profiling was measured using LC-MS between COVID-19 patients and controls following the analysis flowchart in Figure 2A. Considering the levels of glutamate, aspartate, and arginine showed an obvious difference between plasma and serum, the three AAs were excluded for further analysis (Supplementary Table S5). To consider our small sample size, we kept both the plasma and serum results to avoid the selection bias. The difference between sample numbers in COVID-H group by keeping the 15 subjects with two aliquot samples or not was compared. There are no significant difference for the targeted metabolites (Supplementary Table S6). The comparison among different subgroups, including Control *versus* COVID-H and Control old *versus* COVID-H, are quite similar which confirmed the feasibility of our method (Supplementary Table S7).

The levels of AAs, including BCAAs (valine, leucine, and isoleucine) and aromatic AAs (AAAs: tyrosine, phenylalanine, and tryptophan) were increased significantly in COVID-H compared with controls (Table 2, Figure 2B,C,E). The levels of these AAs in the convalescent subjects showed the tendency back to normal, with statistical significance for glutamine, cysteine, phenylalanine, and tryptophan. Accordingly, the catabolic products of BCAAs, acylcarnitines (ACs) presented the similar pattern in the COVID-H and get recovered in the COVID-R group (Figure 2F).

**Table 2:** Concentration of detected AAs in different groups

ConC (μM)	Control (*n*=48)	Con-Old (*n*=32)	Con-Young (*n*=16)	COVID-H (*n*=45)	COVID-R (*n*=21)	*P*-value^a^	*P*-value^b^	*P*-value^c^	*P*-value^d^
Ala	457.35 ± 13.48	453.22 ± 14.94	465.63 ± 27.89	462.2 ± 23.88	518.86 ± 32.3	0.860	0.090	0.751	0.238
Ser	125.41 ± 4.99	129.53 ± 6.37	117.16 ± 7.72	171.61 ± 9.11	135.21 ± 9.19	**0.000**	0.315	**0.000**	0.158
Pro	175.88 ± 7.72	177.56 ± 11.09	172.5 ± 7.11	165.73 ± 5.96	173.05 ± 10.7	0.305	0.837	0.352	0.968
Val	250.5 ± 8.86	247.19 ± 11.32	257.13 ± 14.27	306.28 ± 15.82	280.29 ± 21.1	**0.003**	0.204	**0.003**	0.370
Thr	121.21 ± 3.82	124.08 ± 4.69	115.49 ± 6.58	165.84 ± 11.98	136.25 ± 10.27	**0.001**	0.182	**0.002**	0.098
Lys	171.63 ± 5.25	171.69 ± 6.85	171.5 ± 8.08	212.42 ± 12.86	183.52 ± 12.52	**0.005**	0.389	**0.007**	0.426
Met	17.32 ± 0.74	18.36 ± 0.88	15.24 ± 1.22	12.83 ± 0.53	14.94 ± 1.29	**0.000**	0.094	**0.000**	0.870
His	75.14 ± 1.83	73.62 ± 2.4	78.17 ± 2.61	73.44 ± 3.15	81.99 ± 4.26	0.643	0.151	0.964	0.484
Phe	65.25 ± 2.08	67.22 ± 2.87	61.31 ± 2.31	106.29 ± 6.4	63.07 ± 3.59	**0.000**	0.582	**0.000**	0.683
Tyr	57.38 ± 1.99	60.4 ± 2.59	51.34 ± 2.43	72.99 ± 4.29	63.7 ± 4.76	**0.002**	0.231	**0.014**	**0.028**
Cys	39.41 ± 3.3	45.18 ± 4.35	27.86 ± 3.23	75.97 ± 6.27	28.42 ± 2.63	**0.000**	**0.011**	**0.000**	0.893
Gly	342.6 ± 16.24	355.09 ± 21.46	317.63 ± 22.68	402.36 ± 19.3	333.67 ± 22.33	**0.019**	0.756	0.110	0.623
Leu	113.25 ± 4.83	115.08 ± 6.69	109.58 ± 5.71	137.24 ± 6.41	121.26 ± 10.3	**0.003**	0.424	**0.022**	0.329
Ile	66.99 ± 2.98	69.82 ± 4.01	61.35 ± 3.74	80.9 ± 4.61	74.25 ± 7.53	**0.013**	0.379	0.074	0.136
Cit	6.51 ± 0.29	6.62 ± 0.38	6.28 ± 0.43	6.33 ± 0.5	5.01 ± 0.36	0.756	**0.004**	0.643	**0.029**
Orn	0.26 ± 0.01	0.26 ± 0.01	0.27 ± 0.01	0.31 ± 0.02	0.23 ± 0.03	**0.028**	0.234	**0.028**	0.158
Trp	0.35 ± 0.01	0.35 ± 0.02	0.35 ± 0.02	0.45 ± 0.03	0.53 ± 0.03	**0.002**	**0.000**	**0.002**	**0.000**
Gln	12.69 ± 0.42	12.56 ± 0.56	12.94 ± 0.6	14.5 ± 0.48	12.94 ± 0.55	**0.005**	0.733	**0.010**	0.999
Carnitine	50.18 ± 2.32	49.17 ± 2.15	52.19 ± 5.57	48.79 ± 4.18	47.02 ± 3.08	0.773	0.439	0.937	0.424
Betaine	49.18 ± 2.12	46.93 ± 2.52	53.68 ± 3.74	51.76 ± 4.3	46.98 ± 3.06	0.592	0.564	0.336	0.170
Choline	12.59 ± 1.4	12.63 ± 1.86	12.49 ± 2.01	17.12 ± 1.41	9.05 ± 0.62	**0.024**	**0.024**	0.053	0.119
TML	0.81 ± 0.03	0.8 ± 0.04	0.82 ± 0.06	1.26 ± 0.13	0.74 ± 0.07	**0.002**	0.255	**0.002**	0.346
DMG	3.58 ± 0.16	3.51 ± 0.2	3.71 ± 0.29	5.03 ± 0.45	3.59 ± 0.33	**0.004**	0.970	**0.003**	0.791
C2-AC	8.37 ± 0.51	8.92 ± 0.56	7.29 ± 0.99	11.72 ± 1.91	9.85 ± 0.71	0.096	0.104	0.165	0.037
C3-AC	0.35 ± 0.02	0.35 ± 0.03	0.34 ± 0.04	0.41 ± 0.06	0.39 ± 0.04	0.317	0.337	0.345	0.439
C4-AC	0.19 ± 0.01	0.19 ± 0.02	0.2 ± 0.02	0.25 ± 0.04	0.16 ± 0.02	0.185	0.126	0.161	0.105
C5-AC	0.06 ± 0.01	0.06 ± 0.01	0.07 ± 0.01	0.11 ± 0.02	0.11 ± 0.01	**0.010**	**0.000**	**0.006**	**0.010**
C6-AC	0.03 ± 0.01	0.04 ± 0.01	0.02 ± 0.01	0.05 ± 0.01	0.05 ± 0.01	**0.012**	**0.026**	0.051	**0.005**
C8-AC	0.08 ± 0.01	0.09 ± 0.02	0.06 ± 0.01	0.12 ± 0.01	0.16 ± 0.02	**0.040**	**0.000**	0.184	**0.000**
C10-AC	0.16 ± 0.01	0.17 ± 0.02	0.14 ± 0.01	0.20 ± 0.02	0.28 ± 0.03	**0.041**	**0.000**	0.185	**0.000**

ConC: Concentration Keys: Ala, alanine; Ser, serine; pro, Proline; Val, valine; Thr, theronine; Lys, lysine; Met, methionine; His, histidine; Phe, phenylalanine; Tyr, tyrosine; Cys, cysteine; Gly, glycine; Leu, leucine; Ile, isoleucine; Cit, citrulline; Orn, Orthinine; Trp, tryptophan; Gln, glutamine; TML, trimethyllysine; DMG, Dimethylglycine. AC, acylcarnitine; Con, Control; Con-Old, control matched with COVID-H from age and sex; Con-Young, control matched with COVID-R from age and sex; COVID-H, hospitalized subjects of COVID-19; COVID-R, Recovered subjects of COVID-19. All values were presented as mean ± SEM. *P*-value^a^: *P* value between control and COVID-H. *P*-value^b^: *P* value between control and COVID-R. *P*-value^c^: *P* value between control-Old and COVID-H. *P*-value^d^: *P* value between control-Young and COVID-R.

Values in bold denote *P*<0.05.

AAs involved in one-carbon metabolism including serine, glycine, choline, and dimethylglycine (DMG) were also increased significantly, together with the decreased level of methionine in the COVID-H (Figure 2D). The levels of serine and glycine recover in the group of COVID-R, while the decreased levels of methionine and choline in the convalescent group showed significance compared with the controls.

The metabolites involved in the carnitine pathway showed an increased in COVID-H. These changes in medium-chain ACs decreased in COVID-R but they did not recover to the normal levels except for TML, which implied the existence of incomplete fatty acid β-oxidation in the convalescent period.

In a similar way, the FDR-corrected multinomial logistic regression determined significant increase in the levels of serine (*P*=0.006, OR = 1.02), phenylalanine (*P*=0.001, OR = 1.07), cysteine (*P*=0.014, OR = 1.03), and TML (*P*=0.021, OR = 13.04), together with a decreased methionine level (*P*=0.004, OR = 0.76) in COVID-H when compared with controls (Supplementary Table S8). The increased level of methionine and decreased level of TML showed significance in the convalescent subjects when compared with hospitalized patients. Notably, the levels of aromatic tyrosine were increased significantly in differentiating COVID-R and control group when considering the confound factors.

### Multivariate data analysis of the metabolic and biochemical profiling dataset

The multivariate analysis, containing metabolic and biochemistry profile, revealed a distinct pattern associated with infectious status (Supplementary Figure S1A–C). The cluster tree plot based on the fold change ratio relative to control showed COVID-R patients were more close to the control group, regardless of the metabolic or biochemical profile (Supplementary Figure S2A,B). It indicated that the metabolic and biochemical dysfunction in COVID-R is recovering compared with COVID-H.

**Figure 3:**
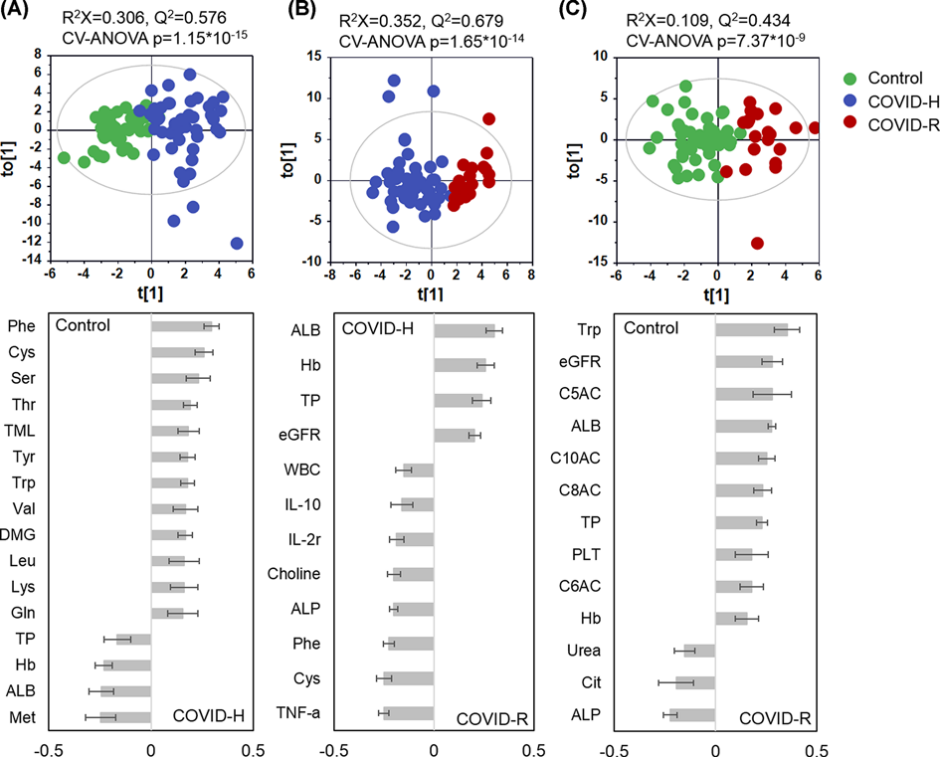
Scores plot and its corresponding w* plot for differentiating each paired group Variables with weight values higher than 0.15 were regarded as contributed to the group separation. (**A**) O-PLS-DA scores plot and w* plot for the comparison between hospitalized patients and its corresponding control group. R^2^ = 0.306, Q^2^ = 0.576, *P*_CV-ANOVA_=1.15 × 10^−15^; (**B**) O-PLS-DA scores plot and w* plot for the comparison between hospitalized patients and recovered subjects. R^2^ = 0.352, Q^2^ = 0.679, *P*_CV-ANOVA_=1.65 × 10^−14^; (**C**) O-PLS-DA scores plot and w* plot for the comparison between convalescent subjects and its corresponding control group. R^2^ = 0.109, Q^2^ = 0.434, *P*_CV-ANOVA_=7.37 × 10^−9^. Keys: Con, Control (●); COVID-H, hospitalized subjects of COVID-19 (●); COVID-R, Recovered subjects of COVID-19 (●).

The O-PLS-DA models were performed for exploring variables contributed to compared two groups (Figure 3). The predictable Q^2^ value and a significant *P-*value from CV-ANOVA revealed an obvious separation between every two-group comparison, indicating distinct metabolic pattern in different infectious status. For the validated models, variables with weight values higher than 0.15 were regarded as significant. Ten AAs and one-carbon metabolites (serine, TML) showed a clear increase in COVID-H when compared with controls, together with the decreased levels for TP, Hb, ALB, and methionine (Figure 3A). The cytokine factors, such as IL-2, IL-10, and TNF-α presented higher expression in group COVID-H when compared with COVID-R (Figure 3B). Notably, models between patients at the convalescent stage and control were also validated by CV-ANOVA. The ACs-related metabolites, including increased C5-AC, C6-AC, C8-AC, and C10-AC, together with decreased levels of citrulline (Cit) and ALP in COVID-R when compared with controls (Figure 3C), indicated the existence of incomplete fatty acid β-oxidation in the convalescent period [11]. Furthermore, the comparison between COVID-H, COVID-R with their aged-matched control (Control-Old and Control-Young) showed similar metabolic patterns when compared with those of all control samples (Supplementary Figure S3A,B). These results revealed that metabolic and biochemical profiling dataset can distinguish different metabolism patterns in diverse infectious status. Most of the metabolic dysfunction may be recovered in 1-month convalescent stage except for the AC-related metabolites, which might imply the virus-induced long-term effects on patients.

## Discussion

The COVID-19 pandemic often reported liver injury apart from the pulmonary symptom. In the present study, we utilized a combination of targeted AA profile and clinical biochemical profile to analyze the plasma of COVID-19 subjects at the hospitalization stage and 1-month post-infection convalescent stage, respectively. We presented several findings. First, the SARS-CoV-2 virus led to stimulated systemic inflammatory and reduced liver synthesis capacity in COVID-19 subjects, which manifested the disturbed BCAAs, AAAs, and one-carbon AAs metabolism. Second, increased levels of AC related metabolites in the convalescent subjects indicated the long-term effects on patients.

**Figure 4:**
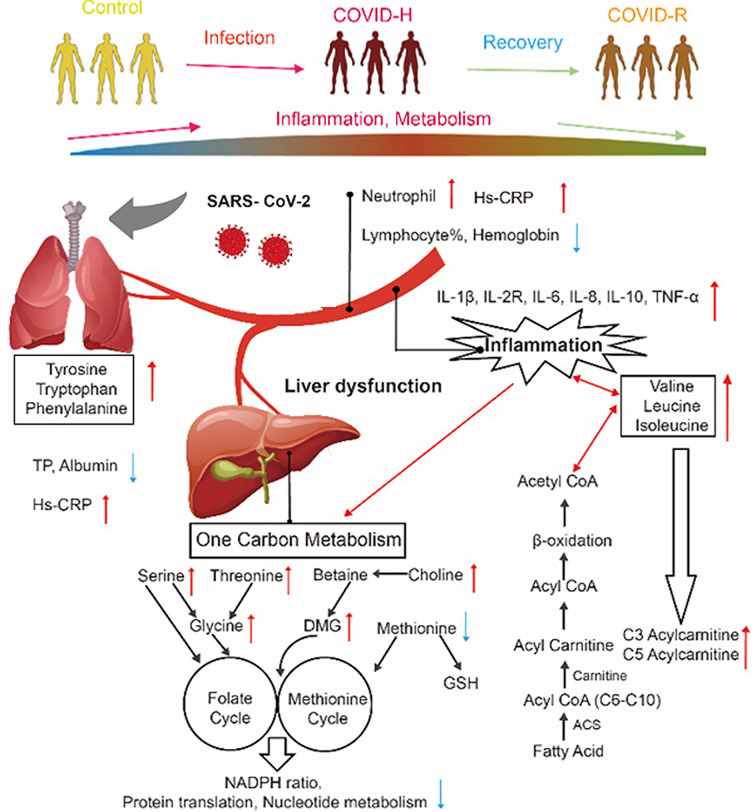
Summarized alteration of metabolic disturbances in hepatic dysfunction induced by the SARS-CoV-2 virus The red arrows indicated the upward index in hospitalized patients. The blue arrows indicated the downward index in hospitalized patients.

In patients with SARS-CoV-2 infection, there are two distinct but overlapped phases: an initial viral response followed by host hyperinflammatory responses [20,21]. The excessive host inflammatory response ultimately leads to vascular damage, immunopathology, and worsening clinical outcomes [22]. In our study, we found immune relative cells (neutrophils) were increased significantly in COVID-H patients and subsequently decreased in the recovery state (Figure 4). Excess neutrophil could be a source of extracellular traps, which may elicit the severe multiorgan consequences [23] and predict poor outcomes in COVID-19 subjects [24]. The increasing plasma cytokines (IL-1β/2R/6/8/10 and TNF-α) confirmed the cytokine storm following the virus infection. Besides, the increased IL-6 also could stimulate an acute-phase protein (CRP) in hepatocytes. On the other hand, the omics-driven global metabolomics [17,25] and proteomics [17,26] works confirmed disturbance in the coagulation system and inflammatory modulators in COVID-19 patients.

AA metabolism plays a vital role in physiology and pathophysiology. The increased levels of AA acid profiles, together with the reduced liver total protein synthesis capacity (decreased total protein and ALB) implied the changed AAs come from the protein breakdown instead of the altered AA clearance. In the present study, the specific AAs, including glycine, serine, threonine, choline, DMG that involved in one-carbon metabolism, were increased in severe COVID-19 patients. One-carbon metabolism generates diverse outputs, such as the synthesis of nucleotides, lipids, and proteins; the maintenance of redox balance; and the substrates for methylation reactions [16]. A previous study found one-carbon metabolism intermediate S-adenosylmethionine (SAM) plays as a key metabolite in supporting LPS-induced inflammation via direct communication between the ‘metabolic state’ and the ‘chromatin state’ during inflammation [27]. And targeting SAM generation in pro-inflammatory macrophages represents a potential therapeutic approach to suppress inflammation. However, methionine exerts antioxidant function in animals mainly through the glutathione pathway and the redox pathway [28], was decreased in severe COVID-19 patients, which indicates higher oxidative stress state following SARS-CoV-2 infection.

We also found BCAAs were increased in severe COVID-19 patients and decreased in convalescent patients. Increased BCAAs induced eNOS expression, ROS production, and pro-inflammatory responses through the transcription factor NF-κB in endothelial cells [29,30]. Prospective observational studies also show that higher levels of circulating BCAAs are positively associated with markers of insulin resistance [31] and risk of incident T2DM [32,33]. Recent genetic studies have also implicated the metabolism of BCAAs in the development of diabetes [34,35]. Therefore, the existence of diabetic complications may deteriorate metabolic dysfunction and accelerate SARS-CoV-2 induced liver injury and mortality through BCAA dysfunctional metabolism in patients with diabetes.

The present study has several limitations. First, only 45 samples from 28 patients were collected in the infection group, which might induce clinical bias. Second, even the multinomial logistic regression was adjusted the potential bias in this small cohort, there still have some other symptoms and unforeseen confounders we could not fully correct.

In conclusion, the SARS-CoV-2 induced imbalance of the AA profiling for COVID-19 patients. The majority of disturbed AAs recovered in 1-month while the incomplete fatty acid oxidation products suggested it might take longer time for the patients at the convalescent stage to get complete recovery from this disease.

## Supplementary Material

Supplementary Figures S1-S3 and Tables S1-S8

## Data Availability

The data used to support the findings of the present study are available from the corresponding author upon request.

## Abbreviations

AAA: aromatic amino acid
AA: amino acid
AC: acylcarnitine
ALB: albumin
BCAA: branched-chain AA
COVID-19: coronavirus disease 2019
COVID-H: hospitalized COVID-19 patients
COVID-R: COVID-19 subjects at the recovery stage
CRP: C-reaction protein
CV-ANOVA: cross-validation ANOVA
DMG: dimethylglycine
FDR: false discovery rate
IL: interleukin
LC-MS: liquid chromatography tandem mass spectrometry
O-PLS-DA: Orthogonal Partial Least-Squares-Discriminant analysis
PCA: principal component analysis
PLS-DA: Partial Least-Squares-Discriminant analysis
SAM: S-adenosylmethionine
SARS-CoV-2: severe acute respiratory syndrome coronavirus 2
TML: trimethyllysine
TNF-α: tumor necrosis factor-α
TP: total protein

## References

[1] Yao X.-H., He Z.-C., Li T.-Y., Zhang H.-R., Wang Y., Mou H. et al. (2020) Pathological evidence for residual SARS-CoV-2 in pulmonary tissues of a ready-for-discharge patient. Cell Res. 30, 541–543 10.1038/s41422-020-0318-5 32346074 PMC7186763

[2] Kimhofer T., Lodge S., Whiley L., Gray N., Loo R.L., Lawler N.G. et al. (2020) Integrative modeling of quantitative plasma lipoprotein, metabolic, and amino acid data reveals a multiorgan pathological signature of SARS-CoV-2 infection. J. Proteome Res. 19, 4442–4454 10.1021/acs.jproteome.0c00519 32806897

[3] Bertolini A., van de Peppel I.P., Bodewes F.A.J.A., Moshage H., Fantin A., Farinati F. et al. (2020) Abnormal liver function tests in patients with COVID-19: relevance and potential pathogenesis. Hepatology 72, 1864–1872 10.1002/hep.31480 32702162 PMC7404414

[4] Kovalic A.J., Huang G., Thuluvath P.J. and Satapathy S.K. (2020) Elevated liver biochemistries in hospitalized Chinese patients with severe COVID-19: systematic review and meta-analysis. Hepatology 10.1007/s12072-020-10078-2 PMC7405102 32692464

[5] Bloom P.P., Meyerowitz E.A., Reinus Z., Daidone M., Gustafson J., Kim A.Y. et al. (2020) Liver biochemistries in hospitalized patients with COVID-19. Hepatology 10.1002/hep.31326 32415860

[6] Huang W., Li C., Wang Z., Wang H., Zhou N., Jiang J. et al. (2020) Decreased serum albumin level indicates poor prognosis of COVID-19 patients: hepatic injury analysis from 2,623 hospitalized cases. Sci. China Life Sci. 63, 1678–1687 10.1007/s11427-020-1729-5 32567003 PMC7306450

[7] Kelly B. and Pearce E.L. (2020) Amino assets: how amino acids support immunity. Cell Metab. 32, 154–175 10.1016/j.cmet.2020.06.010 32649859

[8] Locasale Jason W. and Cantley Lewis C. (2011) Metabolic flux and the regulation of mammalian cell growth. Cell Metab. 14, 443–451 10.1016/j.cmet.2011.07.014 21982705 PMC3196640

[9] White P.J. and Newgard C.B. (2019) Branched-chain amino acids in disease. Science 363, 582 10.1126/science.aav0558 30733403 PMC9940269

[10] Newgard C.B., An J., Bain J.R., Muehlbauer M.J., Stevens R.D., Lien L.F. et al. (2009) A branched-chain amino acid-related metabolic signature that differentiates obese and lean humans and contributes to insulin resistance. Cell Metab. 9, 311–326 10.1016/j.cmet.2009.02.002 19356713 PMC3640280

[11] Boschmann M., Engeli S., Moro C., Luedtke A., Adams F., Gorzelniak K. et al. (2010) LMNA mutations, skeletal muscle lipid metabolism, and insulin resistance. J. Clin. Endocrinol. Metab. 95, 1634–1643 10.1210/jc.2009-1293 20130076 PMC2853996

[12] Li P., Yin Y.L., Li D., Kim S.W. and Wu G. (2007) Amino acids and immune function. Br. J. Nutr. 98, 237–252 10.1017/S000711450769936X 17403271

[13] Wu G., Fang Y.Z., Yang S., Lupton J.R. and Turner N.D. (2004) Glutathione metabolism and its implications for health. J. Nutr. 134, 489–492 10.1093/jn/134.3.489 14988435

[14] Grimble R.F. (2006) The effects of sulfur amino acid intake on immune function in humans. J. Nutr. 136, 1660s–1665s 10.1093/jn/136.6.1660S 16702336

[15] de Koning T.J., Snell K., Duran M., Berger R., Poll-The B.-T. and Surtees R. (2003) L-serine in disease and development. Biochem. J. 371, 653–661 10.1042/bj20021785 12534373 PMC1223326

[16] Locasale J.W. (2013) Serine, glycine and one-carbon units: cancer metabolism in full circle. Nat. Rev. Cancer 13, 572–583 23822983 10.1038/nrc3557 PMC3806315

[17] Shen B., Yi X., Sun Y., Bi X., Du J., Zhang C. et al. (2020) Proteomic and metabolomic characterization of COVID-19 patient sera. Cell 182, 59.e15–72.e15 10.1016/j.cell.2020.05.032 32492406 PMC7254001

[18] Liu Z., Tu M.J., Zhang C., Jilek J.L., Zhang Q.Y. and Yu A.M. (2019) A reliable LC-MS/MS method for the quantification of natural amino acids in mouse plasma: method validation and application to a study on amino acid dynamics during hepatocellular carcinoma progression. J. Chromatogr. B Analyt. Technol. Biomed. Life Sci. 1124, 72–81 10.1016/j.jchromb.2019.05.039 31177050 PMC6626562

[19] Zhao M., Zhao L., Xiong X., He Y., Huang W., Liu Z. et al. (2020) TMAVA, a metabolite of intestinal microbes, is increased in plasma from patients with liver steatosis, inhibits γ-butyrobetaine hydroxylase, and exacerbates fatty liver in mice. Gastroenterology 158, 2266–2281 10.1053/j.gastro.2020.02.033 32105727

[20] Ratajczak M.Z. and Kucia M. (2020) SARS-CoV-2 infection and overactivation of Nlrp3 inflammasome as a trigger of cytokine “storm” and risk factor for damage of hematopoietic stem cells. Leukemia 34, 1726–1729 10.1038/s41375-020-0887-9 32483300 PMC7262681

[21] Oberfeld B., Achanta A., Carpenter K., Chen P., Gilette N.M., Langat P. et al. (2020) SnapShot: COVID-19. Cell 181, 954.e951–954.e951 10.1016/j.cell.2020.04.013 32413300 PMC7190493

[22] Mangalmurti N. and Hunter C.A. (2020) Cytokine storms: understanding COVID-19. Immunity S1074-7613, 30272–30277 10.1016/j.immuni.2020.06.017 PMC7321048 32610079

[23] Barnes B.J., Adrover J.M., Baxter-Stoltzfus A., Borczuk A., Cools-Lartigue J., Crawford J.M. et al. (2020) Targeting potential drivers of COVID-19: neutrophil extracellular traps. J. Exp. Med. 217, e20200652 10.1084/jem.20200652 32302401 PMC7161085

[24] Wang D., Hu B., Hu C., Zhu F., Liu X., Zhang J. et al. (2020) Clinical characteristics of 138 hospitalized patients with 2019 novel coronavirus-infected pneumonia in Wuhan, China. JAMA 323, 1061–1069 10.1001/jama.2020.1585 32031570 PMC7042881

[25] Song J.W., Lam S.M., Fan X., Cao W.J., Wang S.Y., Tian H. et al. (2020) Omics-driven systems interrogation of metabolic dysregulation in COVID-19 pathogenesis. Cell Metab. 32, 188.e185–202.e185 10.1016/j.cmet.2020.06.016 32610096 PMC7311890

[26] Messner C.B., Demichev V., Wendisch D., Michalick L., White M., Freiwald A. et al. (2020) Ultra-high-throughput clinical proteomics reveals classifiers of COVID-19 infection. Cell Syst. 11, 11.e14–24.e14 10.1016/j.cels.2020.05.012 32619549 PMC7264033

[27] Yu W., Wang Z., Zhang K., Chi Z., Xu T., Jiang D. et al. (2019) One-carbon metabolism supports s-adenosylmethionine and histone methylation to drive inflammatory macrophages. Mol. Cell 75, 1147.e1145–1160.e1145 10.1016/j.molcel.2019.06.039 31420217

[28] Eriksson S., Prigge J.R., Talago E.A., Arnér E.S. and Schmidt E.E. (2015) Dietary methionine can sustain cytosolic redox homeostasis in the mouse liver. Nat. Commun. 6, 6479 10.1038/ncomms7479 25790857 PMC4369796

[29] Zhenyukh O., González-Amor M., Rodrigues-Diez R.R., Esteban V., Ruiz-Ortega M., Salaices M. et al. (2018) Branched-chain amino acids promote endothelial dysfunction through increased reactive oxygen species generation and inflammation. J. Cell. Mol. Med. 22, 4948–4962 10.1111/jcmm.13759 30063118 PMC6156282

[30] Zhenyukh O., Civantos E., Ruiz-Ortega M., Sánchez M.S., Vázquez C., Peiró C. et al. (2017) High concentration of branched-chain amino acids promotes oxidative stress, inflammation and migration of human peripheral blood mononuclear cells via mTORC1 activation. Free Radic. Biol. Med. 104, 165–177 10.1016/j.freeradbiomed.2017.01.009 28089725

[31] Würtz P., Soininen P., Kangas A.J., Rönnemaa T., Lehtimäki T., Kähönen M. et al. (2013) Branched-chain and aromatic amino acids are predictors of insulin resistance in young adults. Diabetes Care 36, 648–655 10.2337/dc12-0895 23129134 PMC3579331

[32] Wang T.J., Larson M.G., Vasan R.S., Cheng S., Rhee E.P., McCabe E. et al. (2011) Metabolite profiles and the risk of developing diabetes. Nat. Med. 17, 448–453 10.1038/nm.2307 21423183 PMC3126616

[33] Guasch-Ferré M., Hruby A., Toledo E., Clish C.B., Martínez-González M.A., Salas-Salvadó J. et al. (2016) Metabolomics in prediabetes and diabetes: a systematic review and meta-analysis. Diabetes Care 39, 833–846 10.2337/dc15-2251 27208380 PMC4839172

[34] Lotta L.A., Scott R.A., Sharp S.J., Burgess S., Luan J., Tillin T. et al. (2016) Genetic predisposition to an impaired metabolism of the branched-chain amino acids and risk of type 2 diabetes: a Mendelian randomisation analysis. PLoS Med. 13, e1002179 10.1371/journal.pmed.1002179 27898682 PMC5127513

[35] Wang Q., Holmes M.V., Davey Smith G. and Ala-Korpela M. (2017) Genetic support for a causal role of insulin resistance on circulating branched-chain amino acids and inflammation. Diabetes Care 40, 1779–1786 10.2337/dc17-1642 29046328 PMC5701741

