# Supplementary material for: The SARS-CoV-2 induced targeted amino acid profiling in patients at hospitalized and convalescent stage
Source: Biosci Rep. 2021 Mar 10;41(3):BSR20204201. doi: 10.1042/BSR20204201 (PMC7955102; doi:10.1042/BSR20204201)
Supplement: Supplementary Figures S1-S3 and Tables S1-S8 [file BSR-2020-4201_supp.pdf]

Figure S1

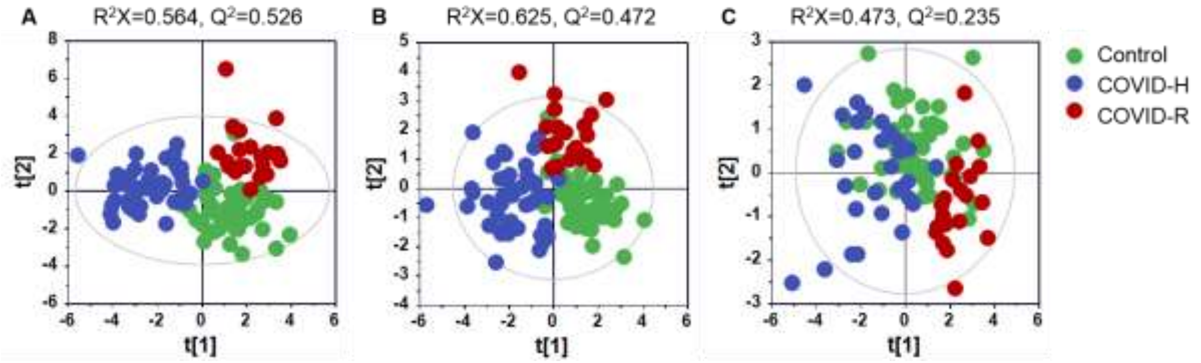

Figure S1. O-PLS-DA plots of the metabolic and clinical biochemical profiling for three groups. (A) O-PLS-DA plot for the combined metabolic and clinical biochemical profiling for three groups. R^2=0.564, Q^2=0.526; (B) O-PLS-DA plot for the metabolic profiling only for three groups. R^2=0.625, Q^2=0.472; (C) O-PLS-DA plot for the clinical biochemical profiling for three groups. R^2=0.473, Q^2=0.235.

Figure S2

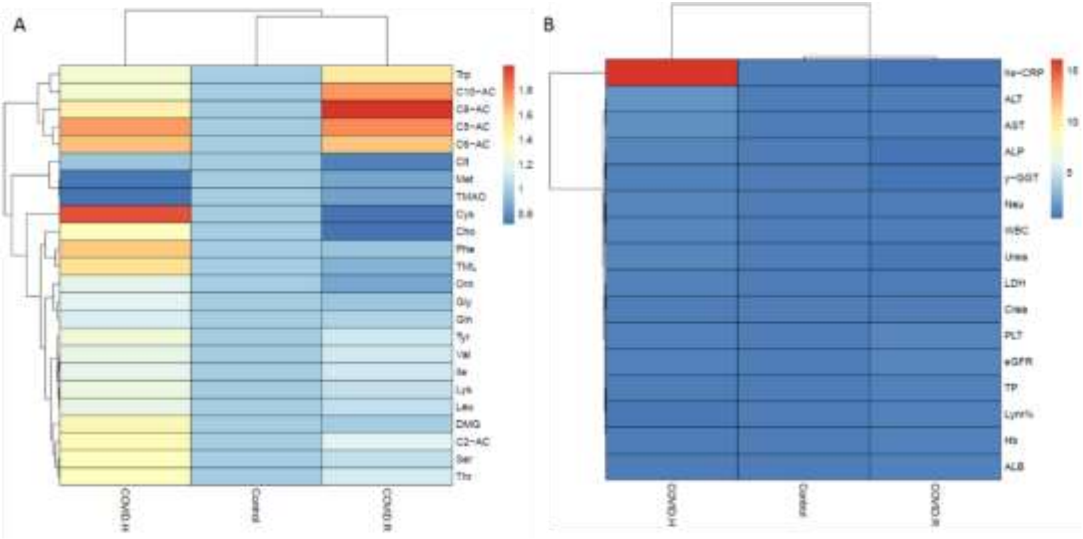

Figure S2. Cluster tree plots of metabolic profiling and biochemistry profiling for three groups. (A) The cluster tree plot is based on the fold change ratio relative to the control group of metabolic profiling for three groups. (B) The cluster tree plot is based on the fold change ratio relative to the control group of biochemistry profiling for three groups.

Figure S3

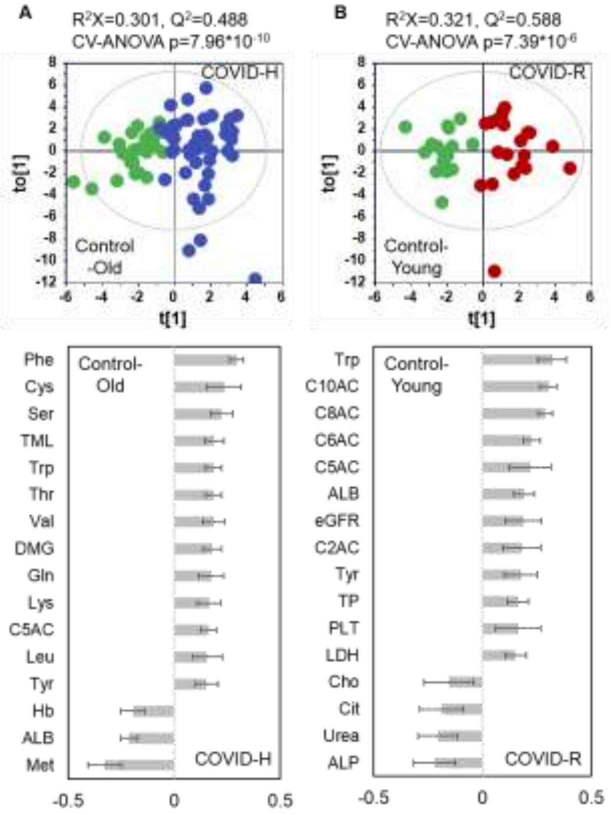

Figure S3. Scores plot and its w* plots for differentiating COVID-H, COVID-R with their aged-matched control, respectively.

Variables with weight values higher than 0.15 were regarded as contributed to the group separation. (**A**) O-PLS-DA scores plot and w* plot for the comparison between hospitalized patients and its matched control-Old group. R^2=0.301, Q^2=0.488, $p_{CV\text{-}ANOVA}$=7.96*10^{-10}; (**B**) O-PLS-DA scores plot and w* plot for the comparison between recovered patients and its matched control-Young subjects. R^2=0.321, Q^2=0.588, $p_{CV\text{-}ANOVA}$=7.39*10^{-6}.

TableS1. Multiple reaction monitoring parameters for targeted metabolites and their internal standards (IS).

Metabolites	Abbreviation	MRM	Internal Standard	MRM (IS)
Phenylalanine	Phe	166.1 → 120.1	IS-Phe (13C, 15N)	176.1 → 129.2
Tryptophan	Trp	205.1 → 146.0	IS-Trp (13C, 15N)	218.1 → 156.2
Leucine	Leu	132.1 → 43.1	IS-Leu (13C, 15N)	139.1 → 46.2
Methionine	Met	150.1 → 104.1	IS-Met (13C, 15N)	156.1 → 109.1
Tyrosine	Tyr	182.1 → 136.0	IS-Tyr (13C, 15N)	192.1 → 145.0
Valine	Val	118.0 → 72.0	IS-Val (13C, 15N)	124.0 → 77.0
Cysteine	Cys	122.0 → 76.0	IS-Cys (13C, 15N)	126.0 → 79.1
Threonine	Thr	120.1 → 74.0	IS-Thr (13C, 15N)	125.1 → 78.0
Glycine	Gly	76.1 → 76.1	IS-Gly (13C, 15N)	78.8 → 78.8
Glutamine	Gln	147.1 → 84.0	IS-Gln (13C, 15N)	154.0 → 89.0
Serine	Ser	106.0 → 60.1	IS-Ser (13C, 15N)	109.9 → 63.1
Lysine	Lys	147.1 → 84.0	IS-Lys (13C, 15N)	155.1 → 90.0
Citrulline	Cit	176.1→70.05	IS-Lys (13C, 15N)	155.1 → 90.0
Ornithine	Orn	133.1→70.1	IS-Lys (13C, 15N)	155.1 → 90.0
Trimethyllysine	TML	189.0→84.0	IS-TML (d9)	198.2→ 84.0
Dimethylglycine	DMG	104.0→ 58.1	IS-DMG (d6)	110.0→ 64.1
Choline	Choline	104.0→ 59.8	IS-Choline (d9)	113.2→ 68.9
Valeryl-L-carnitine	C5- AC	246.1 → 85.0	IS-C5-Carnitine (d3)	249.1 → 85.0
Hexanoyl-L-carnitine	C6-AC	260.0 → 84.8	IS-C6-Carnitine (d3)	263.1 → 84.8
Octanoyl-L-carnitine	C8-AC	288.1 → 85.0	IS-C8-Carnitine (d3)	291.1 → 85.0
Decanoyl-L-carnitine	C10-AC	316.2 → 85.0	IS-C10-Carnitine (d3)	319.2 → 85.0

Table S2: The detailed information of LC gradient condition and targeted MS instrument parameters.

Methods	Amino Acids	Acyl-Carnitines
**LC condition**		
Column	Intrada Amino Acid, 100 * 3 mm	Luna 5u Silica 100A, 2.0*150 mm
Column Chamber T (℃)	35	35
Flow Rate (mL/min)	0.5	0.4
Injection Volume (μL)	3	1
Mobile Phase A	ACN / FA = 100 / 0.3	H_2O / propanoic acid = 100 / 0.1
Mobile Phase B	ACN / 100mM NH_4FA = 20 / 80	MeOH / acetic acid = 100 / 0.1
Gradient (B %)	0.50 min-20%<br>4.50 min-30%<br>5.00 min-60%<br>10.0 min-100%<br>13.0 min-100%<br>13.2 min-20%<br>15.0 min-stop	0.50min-80%<br>3.50min-5%<br>4.50min-5%<br>5.00min-80%<br>6.00min-stop
**MS condition**		
Q TRAP4500	Positive	Positive
Spray voltage (kV)	5.5 ESI+	5.5 ESI+
Source temperature (℃)	600	600
Collision activation parameter	Medium	Medium
Curtain Gas (psi)	35	35
GS1 (psi)	60	60
GS2 (psi)	60	60

Table S3. Concentration of detected clinical biochemical index in all subjects.

Index	Control (N=48)	Con-Old (N=32)	Con-Young (N=16)	COVID-H (N=45)	COVID-R (N=21)
WBC (*10^9/L)	5.84±0.21	5.91±0.27	5.72±0.32	7.12±0.44	5.26±0.27
Neu (%)	62.09±1.23	62.54±1.69	61.19±1.55	64.16±1.88	59.43±1.47

Neu(*10^9/L)	3.66±0.16	3.73±0.22	3.53±0.25	4.8±0.41	3.15±0.2
Lym (%)	28.99±1.11	28.34±1.51	30.3±1.36	23.77±1.67	30.56±1.44
Lym(*10^9/L)	1.67±0.07	1.64±0.09	1.72±0.11	1.56±0.12	1.6±0.1
Hb (g/L)	127.52±2.57	122.63±2.95	137.31±4.06	108.6±3.35	138.1±3.35
PLT(*10^9/L)	199.46±8.59	198.75±10.56	200.88±15.23	214.36±12.6	246.29±16.9
IL-1β (pg/mL)	-	-	-	6.08±1.24	1.31±0.31
IL-2r (U/mL)	-	-	-	880.93±121.72	303.24±23.93
IL-6 (pg/mL)	-	-	-	27.3±7.73	1.01±0.21
IL-8 (pg/mL)	-	-	-	21.88±4.94	8.39±0.51
IL-10 (pg/mL)	-	-	-	6.53±1.29	1.24±0.24
TNF-α (pg/mL)	-	-	-	12.91±1.06	5.35±0.47
ALT (U/L)	21.67±2.35	20.59±2.91	23.81±4.07	36.47±6.74	19.81±3.17
AST(U/L)	22.56±1.81	22.53±2.2	22.63±3.26	34.82±7.5	22.19±1.75
TP (g/L)	71.14±0.79	70.25±0.91	72.92±1.47	66.69±1.25	75.92±0.76
ALB (g/L)	42.16±0.59	41.03±0.7	44.43±0.9	36.9±0.92	46.58±0.44
ALP (U/L)	74.33±4.81	81.88±6.57	59.25±3.99	90.29±7.43	48.1±1.91
γ-GGT (U/L)	42.54±11.93	48.5±17.68	30.63±5.68	47.09±5.95	26.76±3.22
LDH (U/L)	211.9±9.91	239.57±10.99	147.33±6.58	234.33±18.41	189.81±6.57
hs-CRP (mg/L)	1.49±0.31	1.84±0.41	0.32±0.02	24±7.69	0.8±0.22
Urea (mmol/L)	4.96±0.26	5.04±0.37	4.81±0.25	5.97±0.65	4.01±0.2
Crea (umol/L)	66.38±1.9	64.34±2.19	70.44±3.51	62.44±5.62	61.86±3.89
eGFR (ml/min/L)	92±2.71	87.82±2.86	100.36±5.32	93.4±3.27	114.71±3.75

Keys: WBC, white blood cell; Neu, neutrophil; Lym, lymphocyte; Hb, Hemoglobin; PLT, Platelets; IL1β, Inter Leukin 1β; IL2r, Inter Leukin 2r; IL6, Inter Leukin 6; IL8, Inter Leukin 8; IL10 Inter Leukin 10; TNF-α, tumor necrosis factor-alpha; ALT, alanine aminotransferase; AST, aspartate aminotransferase; TP, Total protein; ALB, Albumin; ALP, alkaline phosphatase ; γ-GGT, gamma-glutamyl transpeptidase; LDH, lactate dehydrogenase; hs CRP, hyper sensitive C reaction protein; Crea, creatinine; EGFR, esti mated glomerularfiltrationrate.Con, Control; Con-Old, control matched with COVID-H from age and sex; Con-Young, control matched with COVID-R from age and sex; COVID-H, hospitalized subjects of COVID-19; COVID-R, Recovered subjects of COVID-19. All data was presented as mean ± SEM.

Table S4. Cross-sectional association of clinical biochemistry index in COVID-19 patients.

	Con vs COVID-H OR (95% CI)	p value	FDR adjusted p value	Con vs COVID-R OR (95% CI)	p value	FDR adjusted p value	COVID-H vs COVID-R OR (95% CI)	p value	FDR adjusted p value
TP	**0.75 (0.63,0.88)**	**0.001**	**0.002**	**1.33 (1.08,1.63)**	**0.008**	**0.020**	**0.56 (0.43,0.73)**	**0.000**	**0.000**
ALB	**0.75 (0.62,0.9)**	**0.002**	**0.003**	**1.41 (1.07,1.86)**	**0.014**	**0.024**	**0.53 (0.39,0.73)**	**0.000**	**0.001**
CRP	**1.83 (1.23,2.72)**	**0.003**	**0.003**	1.53 (0.9,2.62)	0.118	0.118	1.19 (0.75,1.9)	0.461	0.544
IL1β	---	---	---	---	---	---	**1.66 (1.11,2.48)**	**0.013**	**0.025**
IL2r	---	---	---	---	---	---	1 (1,1.01)	0.207	0.337
IL6	---	---	---	---	---	---	**2.96 (1.5,5.85)**	**0.002**	**0.006**
IL8	---	---	---	---	---	---	**1.22 (1.06,1.41)**	**0.005**	**0.012**
IL10	---	---	---	---	---	---	**1.91 (1.16,3.16)**	**0.012**	**0.025**
TNF-α	---	---	---	---	---	---	**3.12 (1.55,6.27)**	**0.001**	**0.006**

Abbreviations: Hb, Hemoglobin; PLT, Platelets; TP, Total protein; ALB, Albumin; CRP, C Reaction Protein; EGFR, esti mated glomerularfiltrationrate; IL1b, Inter Leukin 1b; IL2r, Inter Leukin 2r; IL6, Inter Leukin 6; IL8, Inter Leukin 8; IL10 Inter Leukin 10; TNF-a, tumor necrosis factor-alpha.

*Statistically significant associations that passed FDR correction are bolded.

Odds ratios and P values were obtained from logistic regressions. Models were corrected for age, sex, disease history of diabetes, hypertension, coronary heart disease, stroke, Cardiomyopathy, hepatitis and chronic obstructive pulmonary disease.

Table S5. Concentration difference of targeted metabolites between plasma and serum of the same subject.

Concentration (uM)	Plasma (N=15)	Serum (N=15)	*p* value
Ala	428.27±31.24	466.73±55.57	0.552
Ser	149.75±16.03	185.8±19.02	0.158
Pro	178.13±8.16	164.99±13.31	0.407
Val	345.67±24.33	320.53±33.21	0.546
Thr	188.47±21.18	178.97±25.16	0.775
Lys	234±17.54	228.11±31.46	0.872
Glu	56.71±6.03	101.59±11.55	**0.002**
Met	13.67±0.89	12.49±1.07	0.406
His	74.29±4.41	70.14±6.32	0.595
Phe	97.72±7.96	116.51±10.86	0.174
Arg	61.69±7.99	97.79±11.48	**0.015**
Tyr	79.61±6.4	73.34±8.09	0.548
Cys	87.61±9.19	89.05±12.41	0.926
Gly	332.33±26.78	401.93±39.42	0.155
Leu	149.98±10.11	145.75±13.89	0.807
Ile	96.02±7.31	87.53±9.19	0.476
Cit	6.76±1.05	6.05±1.02	0.629
Orn	0.33±0.04	0.27±0.04	0.302
Asp	0.11±0.01	0.24±0.03	**0.001**
Trp	0.47±0.05	0.46±0.06	0.896
Gln	15.02±0.7	14.93±1.17	0.949
Carnitine	52.53±9.2	45.05±5.83	0.498
Betaine	51.97±5.62	45.75±6.01	0.455
Choline	15.15±1.97	18.64±3.48	0.390
TML	1.45±0.27	1.14±0.19	0.362
DMG	5.97±0.85	4.84±0.8	0.344
C2-Acylcarnitine	15.27±4.95	10.56±1.86	0.384
C3-Acylcarnitine	0.49±0.13	0.41±0.1	0.592
C4-Acylcarnitine	0.3±0.09	0.24±0.07	0.626
C5-Acylcarnitine	0.13±0.03	0.11±0.04	0.655
C6-Acylcarnitine	0.06±0.02	0.05±0.01	0.429
C8-Acylcarnitine	0.13±0.03	0.1±0.02	0.364
C10-Acylcarnitine	0.22±0.03	0.19±0.02	0.415

Keys: Ala, alanine; Ser, serine; pro, Proline; Val, valine; Thr, theronine; Lys, lysine; Met, methionine; His, histidine; Phe, phenylalanine; Tyr, tyrosine; Cys, cysteine; Gly, glycine; Leu, leucine; Ile, isoleucine; Cit, citrulline; Orn, Orthinine; Trp, trptophan; Gln, glutamine; TML, trimethyllysine; DMG, Dimethylglycine.All values were presented as mean ± SEM. The statistics were got from the pair-wise comparison between plasma and serum (N=15).

Table S6. The comparison between different sample numbers in COVID-H group

	45 samples	30 samples	p value
Ala	462.2±23.9	459.9±23.5	0.949
Ser	171.6±9.1	164.5±9.8	0.607
Pro	165.7±6	166.1±6.2	0.967
Val	306.3±15.8	299.2±17.3	0.767
Thr	165.8±12	159.3±13	0.718
Lys	212.4±12.9	204.6±11.5	0.670
Met	12.8±0.5	13±0.6	0.835
His	73.4±3.2	75.1±3.6	0.734
Phe	106.3±6.4	101.2±7.9	0.616
Tyr	73±4.3	72.8±5.1	0.979
Cys	76±6.3	69.4±6.9	0.495
Gly	402.4±19.3	402.6±21.7	0.994
Leu	137.2±6.4	133±6.7	0.658
Ile	80.9±4.6	77.6±5.2	0.640
Cit	6.3±0.5	6.5±0.6	0.858
Orn	0.3±0.02	0.3±0.02	0.502
Trp	0.5±0.02	0.4±0.02	0.910
Gln	14.5±0.5	14.3±0.4	0.758
Carnitine	48.8±4.2	50.7±5.6	0.785
Betaine	51.8±4.3	54.8±5.7	0.670
Choline	17.1±1.4	16.4±1.2	0.705
TML	1.3±0.1	1.3±0.2	0.791
DMG	5±0.4	5.1±0.5	0.894
C2-Acylcarnitine	11.7±1.9	12.3±2.7	0.858
C3-Acylcarnitine	0.4±0.1	0.4±0.1	0.982
C4-Acylcarnitine	0.2±0.01	0.3±0.01	0.957
C5-Acylcarnitine	0.1±0.01	0.1±0.01	0.964
C6-Acylcarnitine	0.1±0.01	0.1±0.01	0.898
C8-Acylcarnitine	0.1±0.01	0.1±0.01	0.759
C10-Acylcarnitine	0.2±0.01	0.2±0.01	0.784

Keys: Ala, alanine; Ser, serine; pro, Proline; Val, valine; Thr, theronine; Lys, lysine; Met, methionine; His, histidine; Phe, phenylalanine; Tyr, tyrosine; Cys, cysteine; Gly, glycine; Leu, leucine; Ile, isoleucine; Cit, citrulline; Orn, Orthinine; Trp, trptophan; Gln, glutamine; TML, trimethyllysine; DMG, Dimethylglycine.All values were presented as mean ± SEM. The statistics were got from the unpaired t test comparison between different sample numbers.

Table S7. The comparison among different subgroups in COVID-H group and its control

	Con (N=48)	Con-Old (N=32)	COVID-H (N=45)	Con vs CH	Con-Old vs CH	Con (N=48)	Con-Old (N=32)	COVID-H (N=30)	Con vs CH	Con-Old vs CH
Ala	457.35±13.48	453.22±14.94	462.2±23.88	0.860	0.751	457.35±13.48	453.22±14.94	459.93±23.49	0.925	0.810
Ser	125.41±4.99	129.53±6.37	171.61±9.11	0.000	0.000	125.41±4.99	129.53±6.37	164.51±9.81	0.001	0.004
Pro	175.88±7.72	177.56±11.09	165.73±5.96	0.305	0.352	175.88±7.72	177.56±11.09	166.1±6.17	0.374	0.371
Val	250.5±8.86	247.19±11.32	306.28±15.82	0.003	0.003	250.5±8.86	247.19±11.32	299.15±17.26	0.016	0.015
Thr	121.21±3.82	124.08±4.69	165.84±11.98	0.001	0.002	121.21±3.82	124.08±4.69	159.28±13.02	0.008	0.015
Lys	171.63±5.25	171.69±6.85	212.42±12.86	0.005	0.007	171.63±5.25	171.69±6.85	204.57±11.45	0.012	0.017
Met	17.32±0.74	18.36±0.88	12.83±0.53	0.000	0.000	17.32±0.74	18.36±0.88	13±0.6	0.000	0.000
His	75.14±1.83	73.62±2.4	73.44±3.15	0.643	0.964	75.14±1.83	73.62±2.4	75.09±3.57	0.990	0.734
Phe	65.25±2.08	67.22±2.87	106.29±6.4	0.000	0.000	65.25±2.08	67.22±2.87	101.18±7.88	0.000	0.000
Tyr	57.38±1.99	60.4±2.59	72.99±4.29	0.002	0.014	57.38±1.99	60.4±2.59	72.82±5.11	0.008	0.0357
Cys	39.41±3.3	45.18±4.35	75.97±6.27	0.000	0.000	39.41±3.3	45.18±4.35	69.43±6.92	0.000	0.005
Gly	342.6±16.24	355.09±21.46	402.36±19.3	0.019	0.110	342.6±16.24	355.09±21.46	402.57±21.75	0.028	0.126
Leu	113.25±4.83	115.08±6.69	137.24±6.41	0.003	0.022	113.25±4.83	115.08±6.69	132.98±6.71	0.017	0.064
Ile	66.99±2.98	69.82±4.01	80.9±4.61	0.013	0.074	66.99±2.98	69.82±4.01	77.58±5.18	0.083	0.241
Cit	6.51±0.29	6.62±0.38	6.33±0.5	0.756	0.643	6.51±0.29	6.62±0.38	6.46±0.57	0.947	0.821
Orn	0.26±0.01	0.26±0.01	0.31±0.02	0.028	0.028	0.26±0.01	0.26±0.01	0.33±0.02	0.006	0.006
Trp	0.35±0.01	0.35±0.02	0.45±0.03	0.002	0.002	0.35±0.01	0.35±0.02	0.45±0.03	0.009	0.010
Gln	12.69±0.42	12.56±0.56	14.5±0.48	0.005	0.010	12.69±0.42	12.56±0.56	14.29±0.43	0.013	0.018
Carnitine	50.18±2.32	49.17±2.15	48.79±4.18	0.773	0.937	50.18±2.32	49.17±2.15	50.67±5.58	0.936	0.803
Betaine	49.18±2.12	46.93±2.52	51.76±4.3	0.592	0.336	49.18±2.12	46.93±2.52	54.77±5.7	0.363	0.215
Choline	12.59±1.4	12.63±1.86	17.12±1.41	0.024	0.053	12.59±1.4	12.63±1.86	16.36±1.23	0.065	0.104
TML	0.81±0.03	0.8±0.04	1.26±0.13	0.002	0.002	0.81±0.03	0.8±0.04	1.31±0.17	0.007	0.007
DMG	3.58±0.16	3.51±0.2	5.03±0.45	0.004	0.003	3.58±0.16	3.51±0.2	5.12±0.55	0.011	0.009
C2-AC	8.37±0.51	8.92±0.56	11.72±1.91	0.096	0.165	8.37±0.51	8.92±0.56	12.3±2.72	0.166	0.233
C3-AC	0.35±0.02	0.35±0.03	0.41±0.06	0.317	0.345	0.35±0.02	0.35±0.03	0.41±0.07	0.401	0.423
C4-AC	0.19±0.01	0.19±0.02	0.25±0.04	0.185	0.161	0.19±0.01	0.19±0.02	0.25±0.05	0.256	0.227
C5-AC	0.06±0.01	0.06±0.01	0.11±0.02	0.010	0.006	0.06±0.01	0.06±0.01	0.11±0.02	0.018	0.012
C6-AC	0.03±0.01	0.04±0.01	0.05±0.01	0.012	0.051	0.03±0.01	0.04±0.01	0.05±0.01	0.044	0.102
C8-AC	0.08±0.01	0.09±0.02	0.12±0.01	0.040	0.184	0.08±0.01	0.09±0.02	0.12±0.02	0.049	0.157
C10-AC	0.16±0.01	0.17±0.02	0.20±0.02	0.041	0.185	0.16±0.01	0.17±0.02	0.21±0.02	0.041	0.167

Table S8. Cross-sectional association of metabolites in COVID-19 patients.

	Con vs COVID-H OR (95% CI)	p value	FDR adjusted p value	Con vs COVID-R OR (95% CI)	p value	FDR adjusted p value	COVID-H vs COVID-R OR (95% CI)	p value	FDR adjusted p value
Ser	**1.02 (1.01,1.03)**	**0.001**	**0.006**	1 (0.98,1.01)	0.550	0.660	1.02 (1,1.03)	0.083	0.091
Met	**0.76 (0.65,0.89)**	**0.001**	**0.004**	1.02 (0.91,1.14)	0.745	0.812	**0.78 (0.65,0.93)**	**0.007**	**0.028**
Phe	**1.07 (1.03,1.11)**	**0.000**	**0.001**	0.98 (0.94,1.02)	0.363	0.484	1.05 (1,1.1)	0.048	0.083
Tyr	1.02 (0.98,1.06)	0.371	0.371	**0.93 (0.89,0.98)**	**0.007**	**0.014**	0.95 (0.9,1)	0.069	0.083
Cys	**1.03 (1.01,1.05)**	**0.005**	**0.014**	0.99 (0.95,1.04)	0.812	0.812	1.02 (0.98,1.07)	0.291	0.291
TML	**13.04 (1.75,96.99)**	**0.012**	**0.021**	3.32 (0.31,35.96)	0.324	0.484	**43.27 (2.99,626.41)**	**0.006**	**0.028**

Abbreviations: Ser, serine; THR, threonine; MET, methionine; PHE, phenylalanine; TYR, tyrosine; CYS, cysteine; TML, trimethyllysine; C5-AC, C5 acyl carnitine; C6-AC, C6 acyl carnitine; C8-AC, C8 acyl carnitine; C10-AC, C10 acyl carnitine.

*Statistically significant associations that passed FDR correction are bolded.

Odds ratios and P values were obtained from logistic regressions. Models were corrected for age, sex, disease history of diabetes, hypertension, coronary heart disease, stroke, Cardiomyopathy, hepatitis and chronic obstructive pulmonary disease.
